# Rapid 3D Reconstruction for Image Sequence Acquired from UAV Camera

**DOI:** 10.3390/s18010225

**Published:** 2018-01-14

**Authors:** Yufu Qu, Jianyu Huang, Xuan Zhang

**Affiliations:** Department of Measurement Technology & Instrument, School of Instrumentation Science & Optoelectronics Engineering, Beihang University, Beijing 100191, China; Hjy448@buaa.edu.cn (J.H.); zhangxuanaj@buaa.edu.cn (X.Z.)

**Keywords:** UAV camera, multi-view stereo, structure from motion, 3D reconstruction, point cloud

## Abstract

In order to reconstruct three-dimensional (3D) structures from an image sequence captured by unmanned aerial vehicles’ camera (UAVs) and improve the processing speed, we propose a rapid 3D reconstruction method that is based on an image queue, considering the continuity and relevance of UAV camera images. The proposed approach first compresses the feature points of each image into three principal component points by using the principal component analysis method. In order to select the key images suitable for 3D reconstruction, the principal component points are used to estimate the interrelationships between images. Second, these key images are inserted into a fixed-length image queue. The positions and orientations of the images are calculated, and the 3D coordinates of the feature points are estimated using weighted bundle adjustment. With this structural information, the depth maps of these images can be calculated. Next, we update the image queue by deleting some of the old images and inserting some new images into the queue, and a structural calculation of all the images can be performed by repeating the previous steps. Finally, a dense 3D point cloud can be obtained using the depth–map fusion method. The experimental results indicate that when the texture of the images is complex and the number of images exceeds 100, the proposed method can improve the calculation speed by more than a factor of four with almost no loss of precision. Furthermore, as the number of images increases, the improvement in the calculation speed will become more noticeable.

## 1. Introduction

Because of the rapid development of the unmanned aerial vehicle (UAV) industry in recent years, civil UAVs have been used in agriculture, energy, environment, public safety, infrastructure, and other fields. By carrying a digital camera on a UAV, two-dimensional (2D) images can be obtained. However, as the requirements have grown and matured, 2D images have not been able to meet the requirements of many applications such as three-dimensional (3D) terrain and scene understanding. Thus, there is an urgent need to reconstruct 3D structures from the 2D images collected from UAV camera. The study of the methods in which 3D structures are generated by 2D images is an important branch of computer vision. In this field, many researchers have proposed several methods and theories [1,2,3,4,5,6,7,8,9,10,11,12,13,14,15,16,17]. Among these theories and methods, the three most important categories are the simultaneous localization and mapping (SLAM) [1,2,3], structure from motion (SfM) [4,5,6,7,8,9,10,11,12,13,14] and multiple view stereo (MVS) algorithms [15,16,17], which have been implemented in many practical applications. As the number of images and their resolution increase, the computational times of the algorithms will increase significantly, limiting them in some high-speed reconstruction applications.

Two major contributions in this paper are methods of selecting key images selection and SfM calculation of sequence images. Key images selection is very important to the success of 3D reconstruction. In this paper, a fully automatic approach to key frames extraction without initial pose information is proposed. Principal Component Analysis (PCA) is used to analyze the correlation of features over frames to automate the key frame selection. Considering the continuity of the images taken by UAV camera, this paper proposes a 3D reconstruction method based on an image queue. To ensure the smooth of two consecutive point cloud, an improved bundle-adjustment named weighted bundle-adjustment is used in this paper. After using a fixed-size image queue, the global structure calculation is divided into several local structure calculations, thus improving the speed of the algorithm with almost no loss of accuracy.

## 2. Literature Review

The general 3D reconstruction algorithm without a priori positions and orientation information can be roughly divided into two steps. The first step involves recovering the 3D structure of the scene and the camera motion from the images. The problem addressed in this step is generally referred to as the SfM problem. The second step involves obtaining the 3D topography of the scene captured by the images. This step is usually completed by generating a dense point data cloud or mesh data cloud from multiple images. The problem addressed in this step is generally referred to as the MVS problem. In addition, the research into Real-time simultaneous localization and mapping (SLAM) and 3D reconstruction of the environment have become popular over the past few years. Positions and orientations of monocular camera and sparse point map can be obtained from the images by using SLAM algorithm.

### 2.1. SfM

The SfM algorithm is used to obtain the structure of the 3D scene and the camera motion from the images of stationary objects. There are many similarities between SLAM and SfM. They both estimate the localizations and orientations of camera and sparse features. Nonlinear optimization is widely used in SLAM and SfM algorithms. Researchers have proposed improved algorithms for different situations based on early SfM algorithms [4,5,6]. A variety of SfM strategies have emerged, including incremental [7,8], hierarchical [9], and global [10,11,12] approaches. Among these methods, a very typical one was proposed by Snavely [13], who used it in the 3D reconstruction of real-world objects. With the help of feature point matching, bundle adjustment, and other technologies, Snavely completed the 3D reconstruction of objects by using images of famous landmarks and cities. The SfM algorithm is limited in many applications because of the time-consuming calculation. With the continuous development of computer hardware, multicore technologies, and GPU technologies, the SfM algorithm can now be used in several areas. In many applications, the SfM algorithm has higher requirements for the computing speed and accuracy. There are several improved SfM methods such as the method proposed by Wu [8,14]. These methods can improve the speed of the structure calculation without loss of accuracy. Among the incremental SfM, hierarchical SfM, and global SfM, the incremental SfM is the most popular strategy for the reconstruction of unordered images. Two important steps in incremental SfM are the feature point matching between images, and bundle adjustment. As the resolution and number of images increase, the number of matching points and parameters optimized by bundle adjustment will increase dramatically. This results in a significant increase in the computational complexity of the algorithm and will make it difficult to use it in many applications.

### 2.2. MVS

When the positions and orientations of the cameras are known, the MVS algorithm can reconstruct the 3D structure of a scene by using multiple-view images. One of the most representative methods was proposed by Furukawa [15]. This method estimates the 3D coordinates of the initial points by matching the difference of Gaussians and Harris corner points between different images, followed by patch expansion, point filtering, and other processing. The patch-based matching method is used to match other pixels between images. After that, a dense point data cloud and mesh data cloud can be obtained. Inspired by Furukawa’s method, some researchers have proposed several 3D reconstruction algorithms [16,17,18] based on depth-map fusion. These algorithms can obtain reconstruction results with an even higher density and accuracy. The method proposed by Shen [16] is one of the most representative approaches. The important difference between this method and Furukawa’s method is that it uses the position and orientation information of the cameras as well as the coordinates of the sparse feature points generated from the structure calculation. The estimated depth maps are obtained from the mesh data generated by the sparse feature points. Then, after depth–map refinement and depth–map fusion, a dense 3D point data cloud can be obtained. An implementation of this method can be found in the open-source software openMVS [16].

Furukawa’s approach relies heavily on the texture of the images. When processing weakly textured images, it is difficult for this method to generate a dense point cloud. In addition, the algorithm must repeat the patch expansion and point cloud filtering several times, resulting in a significant increase in the calculation time. Compared to Furukawa’s approach, Shen’s method directly generates a dense point cloud using depth-map fusion. This method can easily and rapidly obtain a dense point cloud. Considering the characteristics of the problems that must be addressed in this study, we use a method similar to Shen’s approach to generating a dense point data cloud.

### 2.3. SLAM

SLAM mainly consists in the simultaneous estimation of the localization of the robot and the map of the environment. The map obtained by SLAM is often required to support other tasks. The popularity of SLAM is connected with the need for indoor applications of mobile robotics. As the UAV industry rises, SLAM algorithms are widely used in UAV applications. Early SLAM approaches are based on Extended Kalman Filters, Rao-Blackwellised Particle Filters, and maximum likelihood estimation. Without priors, MAP estimation reduces to maximum-likelihood estimation. Most SLAM algorithms are based on iterative nonlinear optimization [1,2]. The biggest problem of SLAM is that some algorithms are easily converging to a local minimum. It usually returns a completely wrong estimate. Convex relaxation is proposed by some authors to avoid convergence to local minima. These contributions include the work of Liu et al. [3]. Kinds of improved SLAM algorithms have been proposed to adapt to different applications. Some of them are used for vision-based navigation and mapping.

## 3. Method

### 3.1. Algorithm Principles

**Figure 1:**
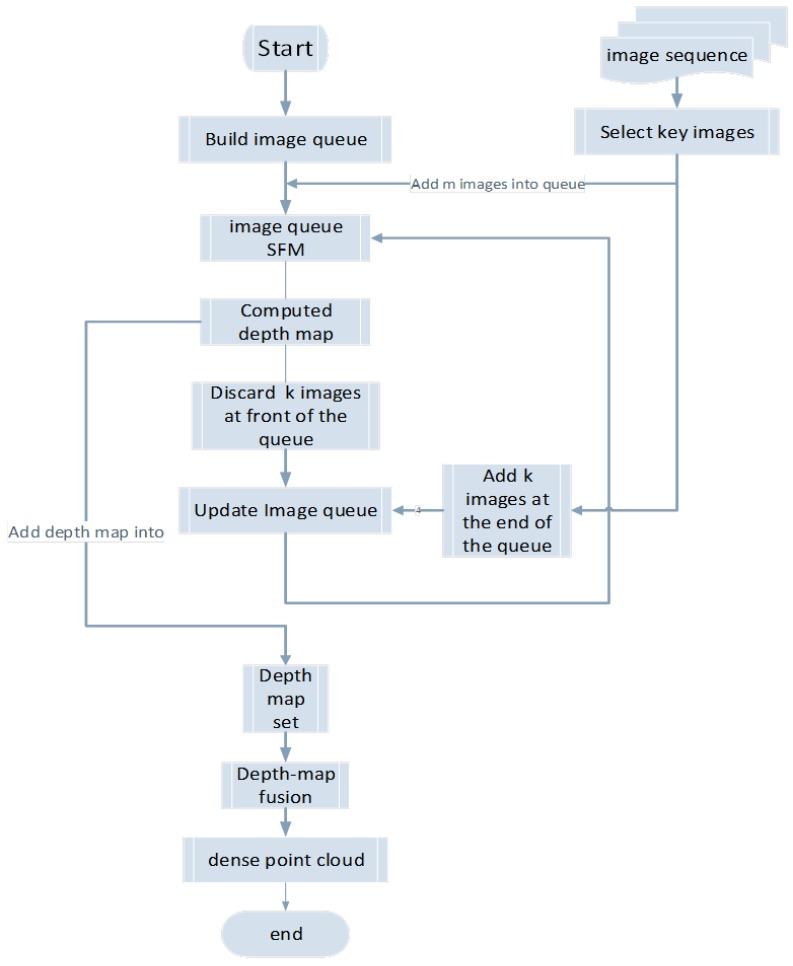
Algorithm flowchart.

The first step of our method involves building a fixed-length image queue, selecting the key images from the video image sequence, and inserting them into the image queue until full. A structural calculation is then performed for the images of the queue. Next, the image queue is updated, several images are deleted from the front of the queue, and the same number of images is placed at the end of the queue. The structural calculation of the images in the queue is then repeated until all images are processed. On an independent thread, the depth maps of the images are calculated and saved in the depth-map set. Finally, all depth maps are fused to generate dense 3D point cloud data. Without the use of ground control points, the result of our method lost the accurate scale of the model. The algorithm flowchart is outlined in Figure 1.

### 3.2. Selecting Key Images

**Figure 2:**
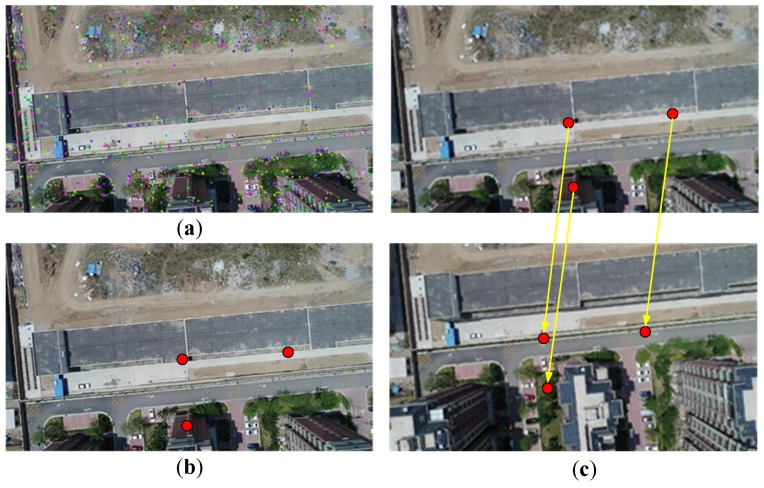
Feature point compression. (**a**) Detecting the feature points of an image; (**b**) calculating the principal component points (PCPs) of the feature points; and (**c**) matching the PCPs.

In order to complete the dense reconstruction of the point cloud and improve the computational speed, the key images (which are suitable for the structural calculation) must first be selected from a large number of UAV video images captured by a camera. The selected key images should have a good overlap of area for the captured scenes. For two consecutive key images, they must meet the key image constraint (denoted as *R* (*I*_1_, *I*_2_)) if they have a sufficient overlap area. In this study, we propose a method for directly selecting key images for reconstructing the UAV camera’s images (the GPS equipped on the UAV can only reach an accuracy on the order of meters; by using GPS information as a reference for the selection of key images, discontinuous images will form). The overlap area between images can be estimated by the correspondence between the feature points of the images. In order to reduce the computational complexity of feature point matching, we propose a method of compressing the feature points based on principal component analysis (PCA). It is assumed that the images used for reconstruction are rich in texture. Three principal component points (PCPs) can be generated from PCA, each reflecting the distribution of the feature points in different images. If the two images are captured almost at the same position, the PCPs of them almost coincide in the same place. Otherwise, the PCPs will move and be located in different positions on the image. The process steps are as follows. First, we use the scale-invariant feature transform (SIFT) [19] feature detection algorithm to detect the feature points of each image (Figure 2a). There must be at least four feature points, and the centroid of these feature points can then be calculated as follows:

(1) $$\bar{p}=\;\frac{1}{n}\sum_{i=1}^{n}P_{i},\;P_{i}=\left(\begin{array}{c} x\\ y \end{array}\right)$$

where *P_i_* is the pixel coordinate of the feature point, and $\bar{p}$ is the centroid. The following matrix is formed by the image coordinates of the feature points:

(2) $$A=\;\left[\begin{array}{c} {\left(P_{1}-\bar{P}\right)}^{T}\\ \vdots \\ {\left(P_{n}-\bar{P}\right)}^{T} \end{array}\right]$$

Then, the singular value decomposition (SVD) of matrix A yields two principal component vectors. The principal component points (PCPs) are obtained from these vectors (Equations (3) and (4)). To compress a large number of feature points into three PCPs (Figure 2b),

(3) $$U\sum V^{*}=svd\left(A\right)$$

(4) $$p_{m1}=\;\bar{P},p_{m2}=\left(V_{1}+\bar{P}\right),p_{m3}=\left(V_{2}+\bar{P}\right)$$

where *p_m_*_1_, *p_m_*_2_, and *p_m_*_3_ are the three PCPs, and *V*_1_ and *V*_2_ are the two vectors of V*. The PCPs can reflect the distribution of the feature points in the image. After that, by calculating the positional relationship of the corresponding PCPs between two consecutive images, we can estimate the overlap area between images. The average displacement ($d_{p}$) between PCPs, as expressed in Equation (5), can be calculated as follows: $d_{p}$ reflects the relative displacement of feature points; when $d_{p}<D_{l}$, it is likely that the two images are almost captured at the same position; and when $d_{p}>D_{h}$, the overlap area of two images becomes too small. In this paper, we use 1/100 of the resolution as the value of $D_{l}$ and 1/10 of the resolution as the value of $D_{h}$. When $d_{p}$ is within the certain range given in Equation (6), the two images will meet the key image constraint $R\left(I_{1},I_{2}\right)$:

(5) $$d_{p}=\frac{1}{3}\sum_{i=1}^{3}\left[{\left(p_{1i}-p_{2i}\right)}^{T}\times (p_{1i}-p_{2i}\right){]}^{0.5}$$

(6) $$R\left(I_{1},I_{2}\right):D_{l}<d_{p}<D_{h}$$

where *p*_1*i*_ is the *i*th PCP of the first image (*I*_1_), and *p*_2*i*_ is that of the second image (*I*_2_). The result is presented in Figure 2c. This is a method for estimating the overlap areas between images, and it is not necessary to calculate the actual correlation between the two images when selecting key images. Moreover, the algorithm is not time-consuming for either the calculation of the PCPs or the estimation of the distance between PCPs. Therefore, this method is suitable for quickly selecting key images from a UAV camera’s video image sequence.

### 3.3. Image Queue SfM

This study focuses on the 3D reconstruction of UAV camera’s images. Considering the continuity of UAV camera’s images, we propose a SfM calculation method based on an image queue. This method constructs a fixed-size image queue and places key images into the queue until full. Then, the structure of the images in the queue is computed, and the queue is updated with new images. Eventually, we will complete the structural calculation of all images by repeating the structural computation and queue update. The image queue SfM includes two steps. The first involves the SfM calculation of the images in the queue. The second involves updating the images in the image queue.

#### 3.3.1. SfM Calculation for the Images in the Queue

**Figure 3:**
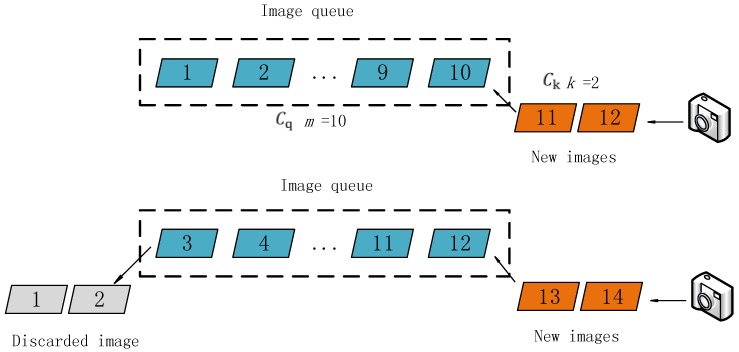
Structure from motion (SfM) calculation of the images in the queue.

We propose the use of the incremental SfM algorithm. The process is illustrated in Figure 3. The collection of all images used for the reconstruction is first recorded as set *C*. The total number of images in *C* is assumed to be *N*. The size of the initial fixed queue is *m* (it is preferred that any two images in the queue have overlapping areas, and *m* can be modified according to the requirements of the calculation speed. When *m* is chosen as a smaller number, the speed increases, but the precision decreases correspondingly). In order to keep the stability of the algorithm, the value of *m* is generally taken greater than 5, and *k* is less than half of *m*. Then, *m* key images are inserted into the image queue. All of the images in the image queue are recorded as *C_q_*, and the structure of all of the images in *C_q_* is calculated.

Considering the accuracy and speed of the algorithm, the SfM approach used in this study uses an incremental SfM algorithm [7]. The steps of the algorithm are summarized below.

1. The SIFT [19] feature detection algorithm is used to detect the feature points on all images in the queue, and the correspondence of the feature points are then obtained by the feature point matching [20] between every two images in the queue.
2. Two images are selected from the queue as the initial image pair using the method proposed in [21]. The fundamental matrix of the two images is obtained by the random sample consensus (RANSAC) method [22], and the essential matrix between the two images is then calculated when the intrinsic matrix (obtained by the calibration method proposed in [23]) is known. The first two terms of radial and tangential distortion parameters are also obtained and used for image rectification. After remapping the pixels onto new locations on the image based on distortion model, the image distortion caused by lens could be eliminated. Then, the positions and orientations of the images can be obtained by decomposing the essential matrix according to [24].
3. According to the correspondence of the feature points in different images, the 3D coordinates of the feature points are obtained by triangulation (the feature points are denoted as $P_{i}\;\left(i=1,\ldots ,t\right)$).
4. The parameters calculated in the previous steps are passed into the bundle adjustment [25] for nonlinear optimization [26].
5. The structure of the initial image pair is calculated, and one of the coordinate systems of the cameras taking the image pair is set as the global coordinate system. The image of the queue that has completed the structure calculation is placed into the set $C_{SFM}$ ($C_{SFM}\subset C_{q}$).
6. The new image ($I_{new}$) is placed into the set ($C_{SFM}$), and the structural calculation is performed. The new image must meet the following two conditions. First, there should be at least one image in $C_{SFM}$ that has common feature points with $I_{new}$. Second, at least six of these common feature points must be in $P_{i}\left(i=1,\ldots ,t\right)$ (in order to improve the stability of the algorithm, this study requires at least 15 common feature points). Finally, all of the parameters from the structure calculation are optimized by bundle adjustment.
7. Repeat step 6 until the structure of all of the images inside the queue is calculated ($C_{SFM}$ = $C_{q}$).

#### 3.3.2. Updating the Image Queue

**Figure 4:**
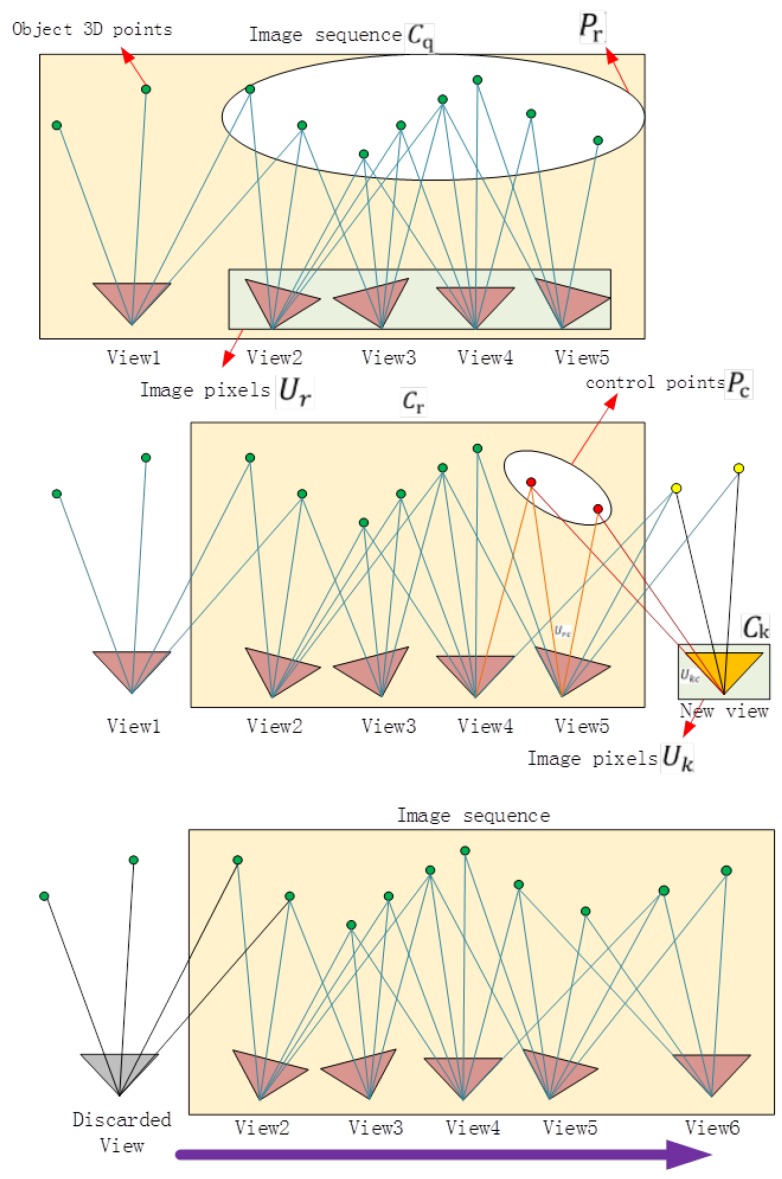
Updating the image queue (*m* = 5, *k* = 1).

After the above steps, the structural calculation of all of the images in $C_{q}$ can be performed. In order to improve the speed of the structural calculation of all of the images in *C*, this study proposes an improved SfM calculation method; the structural calculation of the images is processed in the form of an image queue. Figure 4 illustrates the process of the algorithm. We delete *k* images at the front of the queue, save their structural information, and then place *k* new images at the tail of the queue; these *k* images are then recorded as a set $C_{k}$. The (*m*−*k*) images left in the queue are recorded as a set $C_{r}$($C_{q}=C_{r}\cup C_{k}$), so now $C_{SFM}=\;C_{r}$. The structure of the images in $C_{r}$ is known, and the structural information contains the coordinates of the 3D feature points (marked as $P_{r}$). The corresponding image pixels of *P*_r_ are marked as a set *U_r_*, and the projection relationship is expressed as $P:P_{r}\to U_{r}$. Then, the pixels of the feature points (marked as *U*_k_) of the images in *C*_k_ are detected, and the pixels in *U*_k_ and *U_r_* are matched. We obtain the correspondence $M:U_{rC}↔U_{kc}$$\left(U_{rc}\in U_{r},U_{kc}\in U_{k}\right)$, and *U_rC_* and *U_k_*_c_ are the image pixels of the same object points (marked as $P_{c}$) in different images from *C*_r_ and *C*_k_, respectively, expressed as $P:P_{c}\to U_{kc},P_{c}\to U_{rc},$ where *P*_c_ is the control point. The projection matrix of the images in *C*_k_ can be estimated by the projection relationship between $P_{c}$ and $U_{\mathrm{kc}}$; then, the positions and orientations of the cameras can be calculated. In contrast, $P_{c}$ can be used in the later weighted bundle adjustment to ensure the continuity of the structure. Then, we repeat step 6 until $C_{SFM}=C_{q}$. Finally, the structure of all of the images can be calculated by repeating the following two procedures alternately: calculate the SfM of the images in the queue and update the image queue.

#### 3.3.3. Weighted Bundle Adjustment

**Figure 5:**
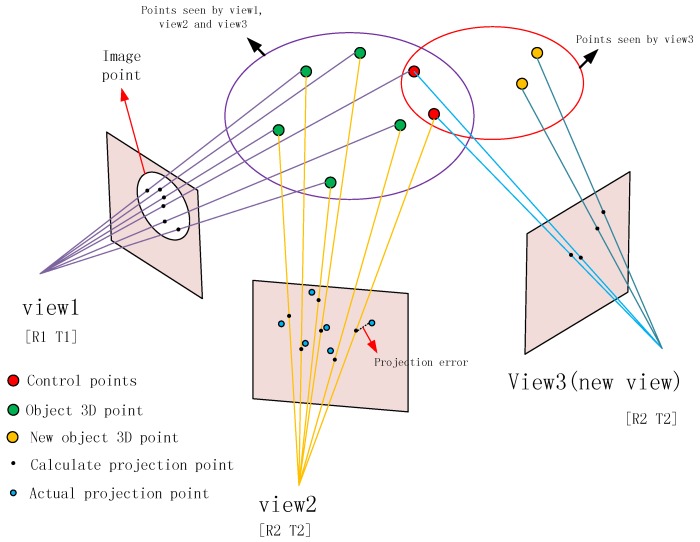
Weighted bundle adjustment.

An important part of the SfM algorithm is bundle adjustment. Our method divides a large number of images into small groups of images in the form of an image queue. When calculating the structure by the queue, optimization of the bundle adjustment causes the parameters to reach the subregion optimum rather than the global optimum. Small differences in the parameters between the subregions will result in discontinuous structures. This problem can be addressed by using control points, which are the points connecting two sets of adjacent feature points of the image, as shown in Figure 5. When we use bundle adjustment to optimize the parameters, we must keep the control points unchanged or with as little change as possible. This is achieved by weighting the error term of the control points. After the first update of the image queue, the formula for the projection error of the bundle adjustment used in step 6 will be altered.

For a single image, Equation (7) is the projection formula of the 3D point to the image pixel, and Equation (8) is the reprojection error formula:

(7) $$\left(\begin{array}{c} v_{i}{}^{f}\\ u_{i}{}^{f} \end{array}\right)=\;K\left[R,t\right]\left(\begin{array}{c} p_{i}\\ 1 \end{array}\right)\;=f\left(R,T,P_{i}\right)$$

(8) $$e_{\mathrm{projrct}}=\sum_{i=1}^{n}\left\{(\left(\begin{array}{c} v_{i}\\ u_{i} \end{array}\right)-{\left(\begin{array}{c} v_{i}{}^{f}\\ u_{i}{}^{f} \end{array}\right))}^{T}\times (\left(\begin{array}{c} v_{i}\\ u_{i} \end{array}\right)-\left(\begin{array}{c} v_{i}{}^{f}\\ u_{i}{}^{f} \end{array}\right))\right\}$$

(9) $$e_{\mathrm{projrct}}=\overset{n}{\underset{i=1}{\sum }}\left\{(\left(\begin{array}{c} v_{i}\\ u_{i} \end{array}\right)-{\left(\begin{array}{c} v_{i}{}^{f}\\ u_{i}{}^{f} \end{array})\right)}^{T}\times (\left(\begin{array}{c} v_{i}\\ u_{i} \end{array}\right)-\left(\begin{array}{c} v_{i}{}^{f}\\ u_{i}{}^{f} \end{array}\right))\right\}+w_{j}\overset{c}{\underset{j=1}{\sum }}\left\{(\left(\begin{array}{c} v_{i}\\ u_{i} \end{array}\right)-{\left(\begin{array}{c} v_{i}{}^{f}\\ u_{i}{}^{f} \end{array})\right)}^{T}\times (\left(\begin{array}{c} v_{i}\\ u_{i} \end{array}\right)-\left(\begin{array}{c} v_{i}{}^{f}\\ u_{i}{}^{f} \end{array}\right))\right\}$$

where *K* is the internal matrix of the camera, *R* and *T* are the external parameters, *P_i_* is the 3D feature point,$\;\left(\begin{array}{c} v_{i}\\ u_{i} \end{array}\right)$ is the actual pixel coordinate of the feature point, and $\left(\begin{array}{c} v_{i}{}^{f}\\ u_{i}{}^{f} \end{array}\right)$ is the pixel coordinate calculated from the structural parameters. The number of control points is *k*. The calculation of the bundle adjustment is a nonlinear least-squares problem. The structural parameters $\left(R,T,P_{i\left(i=1,\ldots ,n\right)}\right)$ can be optimized by minimizing $e_{\mathrm{projrct}}$ after changing the value of the parameters.

The difference between the weighted bundle adjustment and the bundle adjustment is the weight of the control points’ projection error. The weight is $w_{j}$ (after an experimental comparison, a value of 20 is suitable for $w_{j}$). Equation (9) is the reprojection error formula of the weighted bundle adjustment.

#### 3.3.4. MVS

For the dense reconstruction of the object, considering the characteristics of the problem addressed in this study, we use the method based on depth-map fusion to obtain the dense point cloud. The method is similar to that proposed in [16]. The algorithm first obtains the feature points in the structure calculated by the SfM. By using Delaunay triangulation, we can obtain the mesh data from the 3D feature points. Then, the mesh is used as an outline of the object, which is projected onto the plane of the images to obtain the estimated depth maps. The depth maps are optimized and corrected using the pixel matching algorithm based on the patch. Finally, dense point cloud data can be obtained by fusing these depth maps.

## 4. Experiments

### 4.1. Data Sets

**Figure 6:**
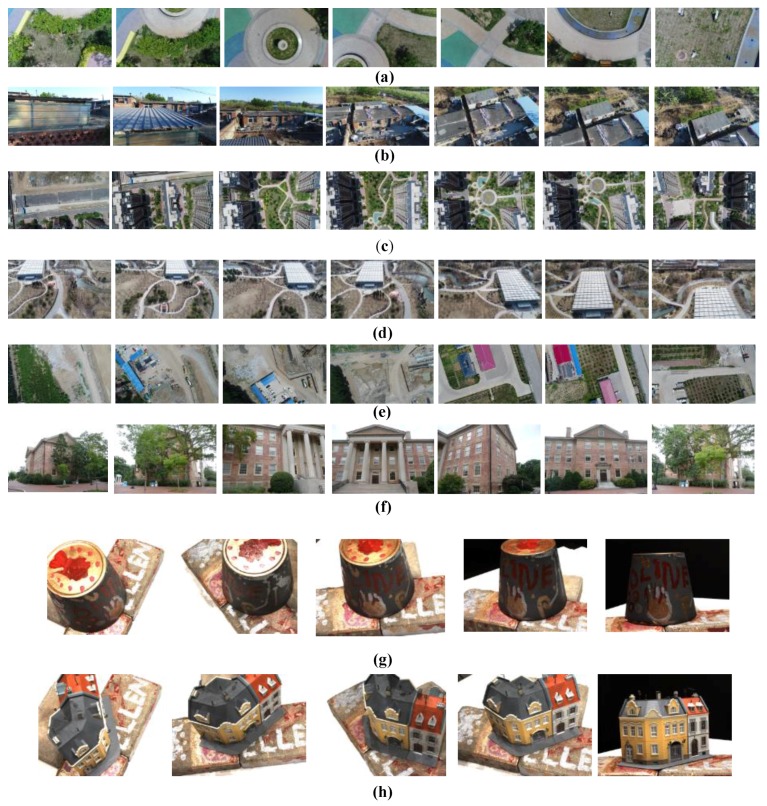
Images for experiment (**a**) Garden; (**b**) Village; (**c**) Building; (**d**) Botanical Garden; (**e**) Factory land; (**f**) Academic building; (**g**) Pot; and (**h**) House.

In order to test the accuracy and speed of the algorithm proposed in this study, real outdoor photographic images taken from a camera fixed on a UAV and standard images together with standard point cloud provided by roboimagedata [27] are used to reconstruct various dense 3D point clouds. The object models and images provide by roboimagedata are scanned with a high precision structured light setup consisting of two Point Grey Research GS3-U3-91S6C-C industrial cameras with resolution of 9.1 Mp and a LG-PF80G DLP projector with a resolution of 1140 × 912 pixels mounted on a rigid aluminum frame. In addition, a high precision New-mark Systems RT-5 turntable is used to provide automatic rotation of the object). Figure 6a–e present some of the outdoor images (different resolution images taken with the same camera) taken from a camera carried by the DJI Phantom 4 Pro UAV (camera hardware: 1/2.3 inch CMOS, Effective 12.4 million pixels. Lens: FOV 94° 20 mm (35 mm format equivalent) f/2.8 Focal point at infinity). Figure 6f presents some images of an academic building taken by a normal digital camera which moves around the building (the camera’s depth of field is near infinity). Figure 6d,e present some of the standard images [28] taken by a camera fixed to a robotic arm (with known positions and orientations) which is provided by roboimagedata. Table 1 lists all of the information for the experimental image data and the parameters used in the algorithm. We used a computer running Windows 7 64-bit with 8 GB of RAM and a quad-core 2.80-GHz Intel (R) Xeon (r) CPU.

### 4.2. Precision Evaluation

**Figure 7:**
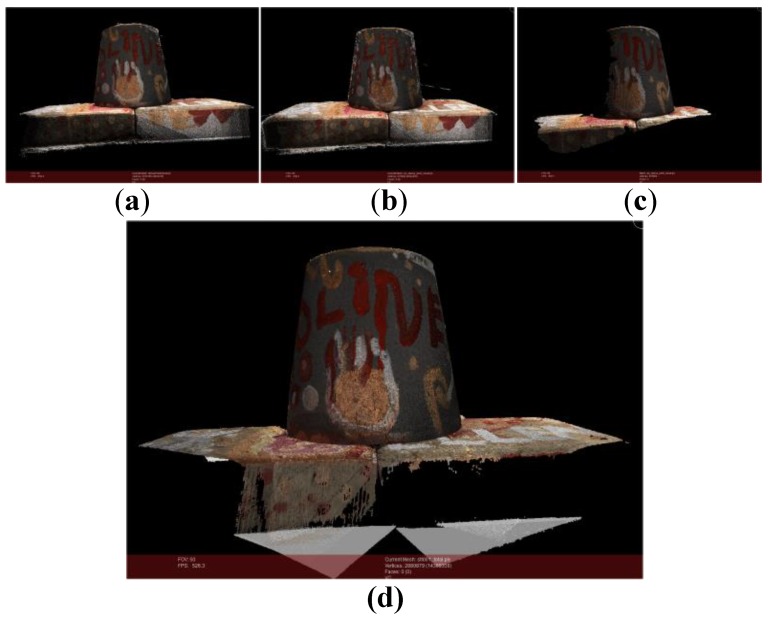
Point cloud comparison. (**a**) Point cloud of our method (*m* = 15, *k* = 6); (**b**) Point cloud of openMVG + openMVS; (**c**) Point cloud of MicMac; (**d**) Standard point cloud.

**Figure 8:**
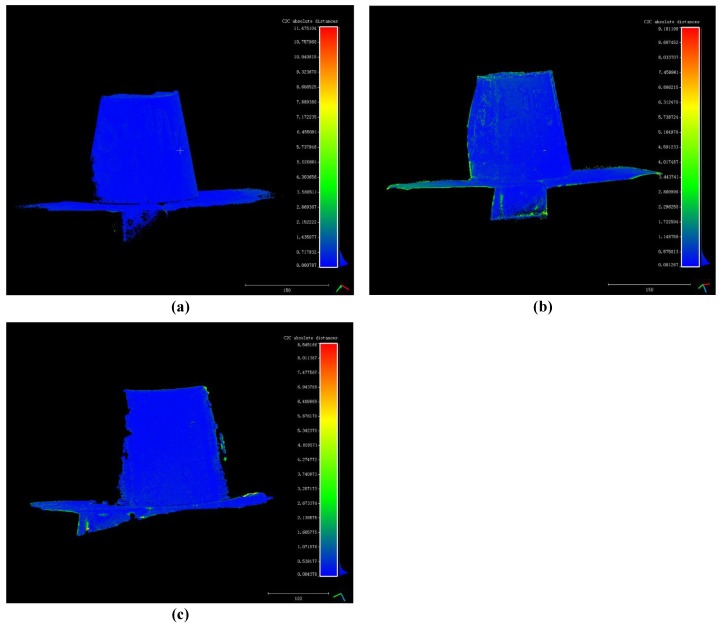
(**a**) Distance point cloud between the proposed method’s result and the standard point cloud; (**b**) Distance point cloud between openMVG + openMVS’s result and the standard point cloud; (**c**) Distance point cloud of MicMac and the standard point cloud.

**Figure 9:**
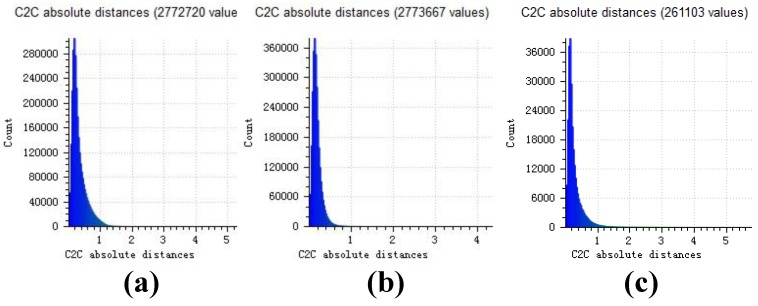
(**a**) Distance histogram of our result; (**b**) Distance histogram of openMVG + openMVS’s result; (**c**) Distance histogram of MicMac’s result.

**Figure 10:**
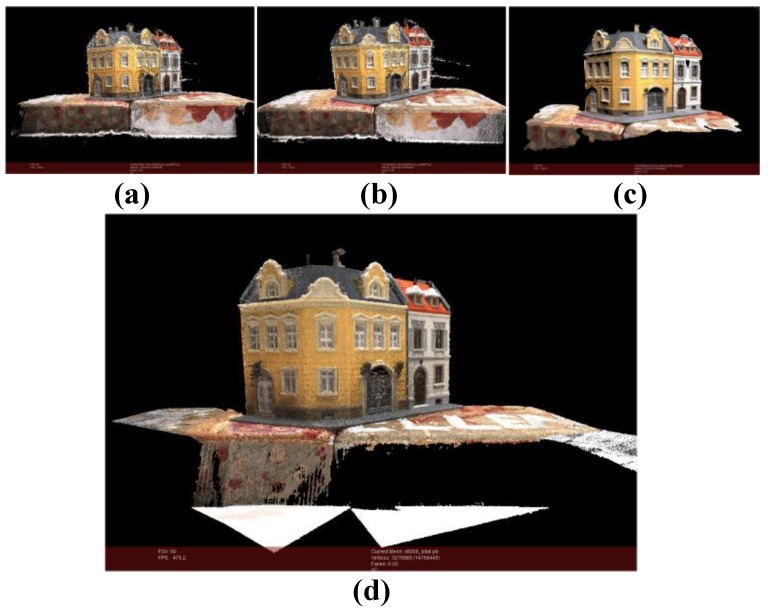
Point cloud comparison. (**a**) Point cloud of our method (*m* = 15, *k* = 6); (**b**) Point cloud of openMVG + openMVS; (**c**) Point cloud of MicMac; (**d**) Standard point cloud.

**Figure 11:**
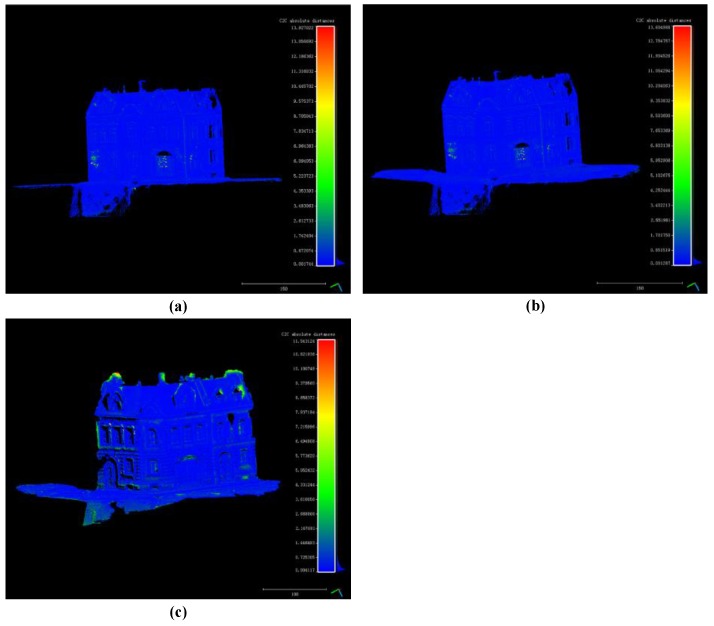
(**a**) Distance point cloud between the proposed method’s result and the standard point cloud; (**b**) Distance point cloud between openMVG + openMVS’s result and the standard point cloud; (**c**) Distance point cloud between MicMac’s result and the standard point cloud.

**Figure 12:**
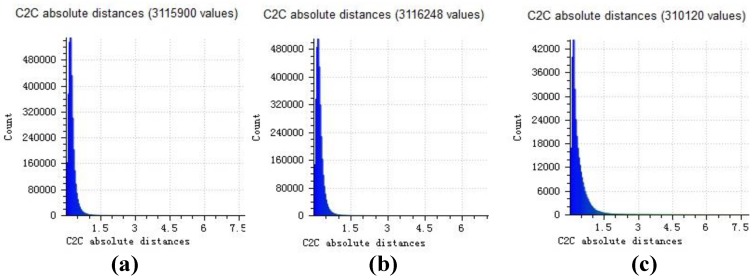
(**a**) Distance histogram of our result; (**b**) Distance histogram of openMVG + openMVS; (**c**) Distance histogram of MicMac.

In order to test the accuracy of the 3D point cloud data obtained by the algorithm proposed in this study, we compared the point cloud generated by our algorithm (PC) with the standard point cloud *PC_STL_* which is captured by structured light scans (The RMS error of all ground truth poses is within 0.15 mm) provided by roboimagedata [27]. The accuracy of the algorithm is determined by calculating the nearest neighbor distance of the two point clouds [28]. First, the position of the point cloud is registered by the iterative nearest point method. For the common part of PC and *PC_STL_*, each point *p*_1_ in the PC, *PC_STL_* is searched for the nearest point *p*_1_’, and the Euclidean distance between *p*_1_ and *p*_1_’ is calculated. The distance point cloud is obtained after the distance calculation of each point and marked with different color. We compare the results of our method to those of openMVG [7], openMVS [16] and MicMac [29,30,31] (three open-source software packages). The main concern of openMVG is SfM calculation, while the main concern of openMVS is dense reconstruction. MicMac is a free open-source photogrammetric suite that can be used in a variety of 3D reconstruction scenarios. They both achieved state-of-the-art results. An open source software named Cloud Compare [32] is used for the test. The results are presented in Figure 7, Figure 8, Figure 9, Figure 10, Figure 11 and Figure 12.

In the first experiment. As shown in Figure 7, point cloud shown in Figure 7a is generated by our method from 49 images (*m* = 15, *k* = 6). The number of points in the point cloud is 2,076,165. Figure 7b the number of points in point cloud of openMVG + openMVS is 2,586,511. Figure 7c the number of points in point cloud generated by MicMac is 270,802. And Figure 7d is standard point cloud provided by roboimagedata. The number of points is 2,880,879.

The distance point clouds are shown in Figure 8a–c. The calculation of distance is performed only on the common part of the two point clouds. Different color means different value of distance.

Distance histograms in Figure 9a–c is statistics results of distance point cloud in Figure 8a–c. For the pot experiment, most distances are less than 1.5 cm when the pot is higher than 200 cm (the relative error is less than 1%).

In the second experiment. As shown in Figure 10, Figure 10a point cloud is generated by our method from 49 images (*m* = 10, *k* = 5). The number of points in the point cloud is 2,618,918. Figure 10b the number of points in Point cloud of openMVG + openMVS is 2,695,354. Figure 10c the number of points in point cloud generated by MicMac is 321,435. And (d) is standard point cloud provided by roboimagedata. The number of points is 3,279,989.

Distance histograms in Figure 12a–c are statistics results of distance point clouds in Figure 11a–c. For the house experiment, most distances are less than 1cm when the house is higher than 150 cm (the relative error is less than 1%).

The number of points of the point clouds generated by our algorithm are almost the same as openMVG + openMVS’s results, and much more than those of MicMac. MicMac’s result is smoother but less dense. The accuracy of our method is almost the same as openMVG + openMVS and MicMac (state-of-the-art methods), but the speed is much faster than them.

### 4.3. Speed Evaluation

In order to test the speed of the proposed algorithm, we compared the time consumed by our method with those consumed by openMVG and MicMac. Different *m* and *k* values for the algorithm are selected, and the same image data are used to run the program under the same hardware conditions. The running times of the algorithm are recorded in Table 2, and the precision is 1 s.

The accuracy of our result is almost the same as result of openMVG and MicMac, but the speed of our algorithm is faster than them. As is shown in Table 2.

There are two aspects that affect the speed of the algorithm. For most feature point matching algorithms, all images must match each other; thus, the time complexity of matching is *O*(*N*^2^). After using the methods proposed in this study, the time complexity becomes $\frac{n}{k}O\left(m\times k\right)$ because the matching calculation occurs only for the images inside the image queue. Although *m* and *k* are fixed and their values are generally much smaller than *N*, the speed of the matching is greatly improved. Second, for the SfM calculations, most of the time is spent on bundle adjustment. Bundle adjustment itself is a nonlinear least-squares problem that optimizes the camera and structural parameters; the calculation time will increase because of the increase in the number of parameters. The proposed method divides the global bundle adjustment, which optimizes a large number of parameters, into several local bundle adjustments so that the number of the parameters remains small and the calculation speed of the algorithm improves greatly.

### 4.4. Results

**Figure 13:**
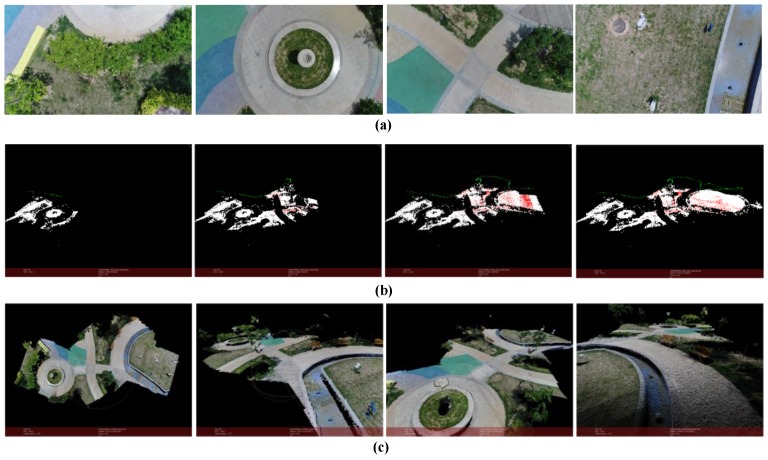
Reconstruction result of a garden. (**a**) Part of the images used for reconstruction; (**b**) Structure calculation of image queue SfM (green points represent the positions of the camera); (**c**) Dense point cloud of the scene.

The result is shown in Figure 13 (*m* = 15, *k* = 6).The scene in this case is captured by an UAV camera in a garden of YanJiao. The flight height is about 15 m from the ground and is kept unchanged. The flight distance is around 50 m. The images’ resolution is 1920 × 1080. And the number of points in point cloud is 4,607,112.

**Figure 14:**
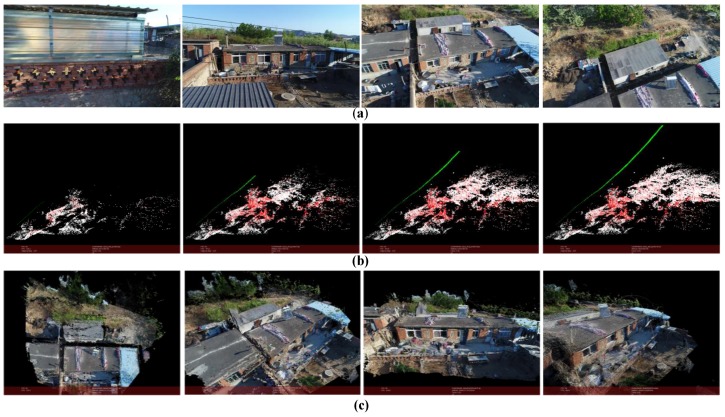
Reconstruction result of a village. (**a**) Part of the images used for reconstruction; (**b**) Structure calculation of image queue SfM (green points represent the positions of the camera); (**c**) Dense point cloud of the scene.

The result is shown in Figure 14 (*m* = 15, *k* = 6). The scene in this case is captured by a UAV camera in a village. The UAV is launched from the ground and flies over the house. The maximum flight height is around 6 m. The flight distance is around 20 m. The images’ resolution is 1920 × 1080. And the number of points in the point cloud is 3,040,551.

**Figure 15:**
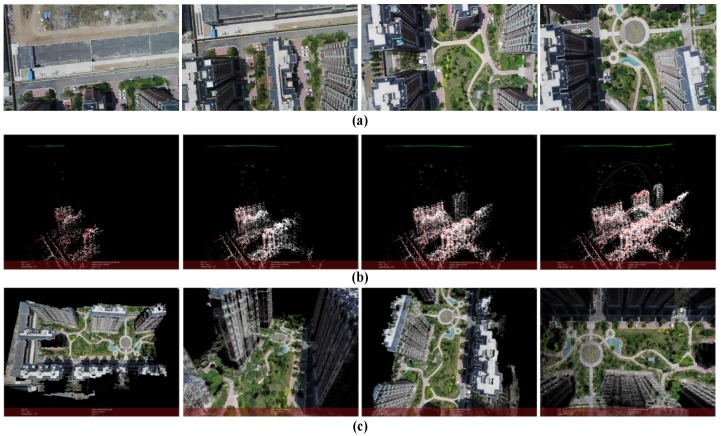
Reconstruction result of buildings. (**a**) Part of the images used for reconstruction; (**b**) Structure calculation of image queue SfM (green points represent the positions of the camera); (**c**) Dense point cloud of the scene.

The result is shown in Figure 15 (*m* = 15, *k* = 6). The scene in this case is captured by a UAV camera in a village. The UAV flight over the top of the buildings. The flight height is around 80 m and is kept unchanged. The flight distance is around 150 m. The images’ resolution is 1280 × 720 and the number of points in point cloud is 2,114,474.

**Figure 16:**
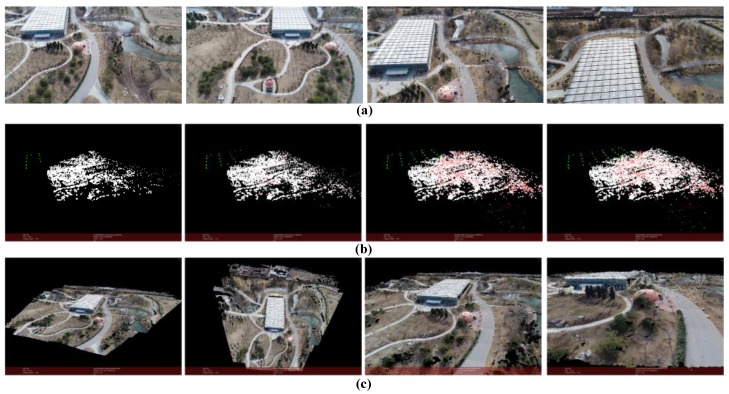
Reconstruction result of botanical garden. (**a**) Part of the images used for reconstruction; (**b**) Structure calculation of image queue SfM (green points represent the positions of camera); (**c**) Dense point cloud of the scene.

The result is shown in Figure 16 (*m* = 10, *k* = 3). In this case, the UAV flight is over a botanical garden. The flight blocks are integrated for many parallel strips. The flight height is around 40 m and kept unchanged. The flight distance is around 50 m. The images’ resolution is 1920 × 1080 and the number of points in point cloud is 2,531,337.

**Figure 17:**
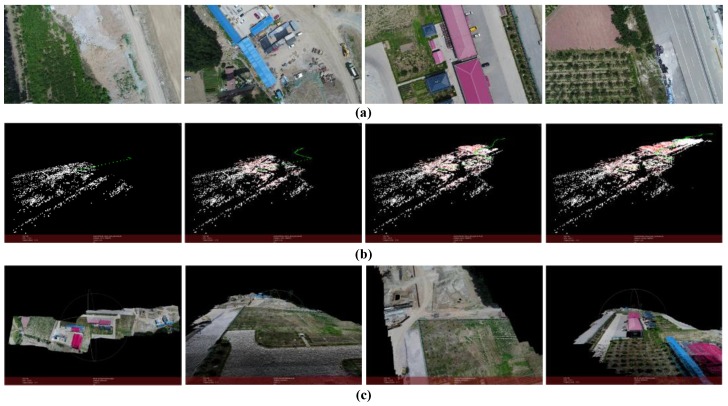
Reconstruction result of botanical garden. (**a**) Part of the images used for reconstruction; (**b**) Structure calculation of image queue SfM (green points represent the positions of camera); (**c**) Dense point cloud of the scene.

The result is shown in Figure 17 (*m* = 20, *k* = 5). In this case, the UAV flight is over a factory land. The flight height is around 90 m and is kept unchanged. The flight distance is around 300 m. The images’ resolution is 1280 × 720 and the number of points in point cloud is 9,021,836.

**Figure 18:**
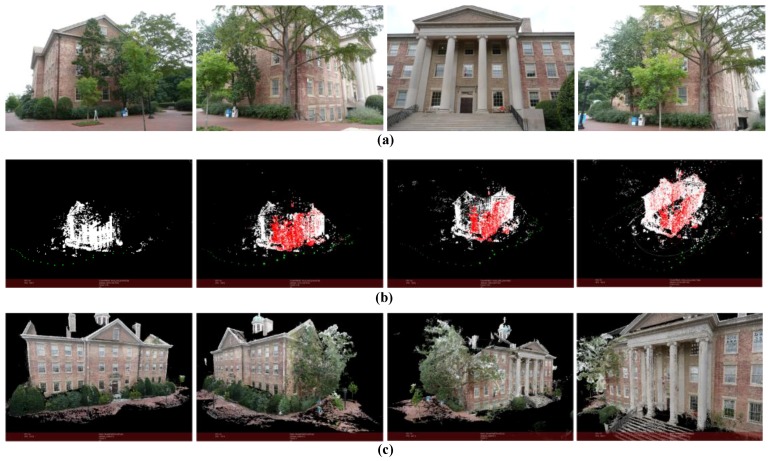
Reconstruction result of botanical garden. (**a**) Part of the images used for reconstruction; (**b**) Structure calculation of image queue SfM (green points represent the positions of camera); (**c**) Dense point cloud of the scene.

The result is shown in Figure 18 (*m* = 25, *k* = 8). In this case, a ground-based camera instead of UAV camera is used to move around the academic building and taken images. The images’ resolution is 1920 × 1080 and the number of points in point cloud is 23,900,173. The result shows that our algorithm can be used in reconstruction from normal digital camera images as long as the images are taken continuously.

The results of experiment images used in this paper are present in Figure 13, Figure 14, Figure 15, Figure 16, Figure 17 and Figure 18. For each example, Figure 18a shows some of the images used for 3D reconstruction. In the Figure 18b four most representative views of SfM, calculation results are selected to present the process of image queue SfM. Green points represent the positions of camera, and red points are control points, white points are structural feature points. Positions and orientations of cameras together with object feature points are derived in the order of camera movement. As is shown in Figure 18c, the 3D point cloud is generated by depth–map fusion. Accurate result can be obtained by using our method as long as the images are captured continuously. The final results accurately reproduce the appearance of the scenes.

## 5. Conclusions

In order to reconstruct the 3D structure of scenes using image sequences, we propose a rapid and accurate 3D reconstruction method based on an image queue. First, a principal component analysis method of the feature points is used to select the key images suitable for 3D reconstruction, which ensures that the algorithm improves the calculation speed with almost no loss of accuracy. Then, considering the continuity and relevance of the UAV camera’s images, we propose a method based on an image queue. Our method divides a global bundle adjustment calculation into several local bundle adjustment calculations, greatly improving the calculation speed of the algorithm and making the structures continuous. Finally, dense 3D point cloud data of the scene are obtained by using depth–map fusion. The experiments demonstrate that when the texture of the images is complex and the number of images exceeds 100, the proposed method can improve the calculation speed by more than a factor of four with almost no loss of calculation accuracy. Furthermore, when the number of images increases, the improvement in the calculation speed will become more noticeable.

When the scene is too long, such as the flight distance is more than 300 m. The structure of the reconstruction will be distorted due to accumulated errors. This problem is solved in global SfM [7] by using loop closure constraint. Our future work will be aimed at cumulative errors elimination and will obtain higher accuracy. With the rise of artificial intelligence research, the parameters of *m* and *k* can be selected automatically by using deep learning and machine learning. Improving the performance of the algorithm in parameter selection is also part of our future work.

## Figures and Tables

**Table 1:** Information for the Experimental Image Data.

Name	Image	Resolution	(*m*, *k*)	$D_{l}$	$D_{h}$
Garden	126	1920 × 1080	(15, 6)(20, 7)(40, 15)	25	150
Village	145	1920 × 1080	(15, 6)(20, 7)(40, 15)	25	150
Building	149	1280 × 720	(15, 6)(20, 7)(40, 15)	20	150
Botanical Garden	42	1920 × 1080	(15, 6)(20, 7)(40, 15)	25	150
Factory Land	170	1280 × 720	(15, 6)(20, 7)(40, 15)	20	150
Academic Building	128	1920 × 1080	(15, 6)(20, 7)(40, 15)	25	150
Pot	49	1600 × 1200	(8, 3)(10, 4)(15, 6)	20	200
House	49	1600 × 1200	(8, 3)(10, 4)(15, 6)	20	200

**Table 2:** Running Time Comparison.

Name	Images	Resolution	Our Method Time (s)			OpenMVG Time (s)	MicMac Time(s)
			*m* = 15, *k* = 6	*m* = 20, *k* = 7	*m* = 40, *k* = 15		
Garden	126	1920 × 1080	284.0	291.0	336.0	1140.0	3072.0
Village	145	1920 × 1080	169.0	209.0	319.0	857.0	2545.0
Building	149	1280 × 720	171.0	164.0	268.0	651.0	2198.0
Botanical Garden	42	1920 × 1080	77.0	82.0	99.0	93.0	243.0
Factory Land	170	1280 × 720	170.0	207.0	343.0	1019.0	3524.0
Academic building	128	1920 × 1080	124.0	182.0	277.0	551.0	4597.0
			***m* = 15, *k* = 6**	***m* = 10, *k* = 4**	***m* = 8, *k* = 3**		
Pot	49	1600 × 1200	35.0	39.0	47.0	56.0	351.0
House	49	1600 × 1200	59.0	53.0	54.0	74.0	467.0
